# What aspects of clinical uncertainty are addressed by published competencies in evidence-based medicine? A scoping review

**DOI:** 10.1136/bmjopen-2025-109013

**Published:** 2026-07-31

**Authors:** Edmund Jack, Jason Hancock, Morwenna Rogers, Karen Mattick

**Affiliations:** 1 GP Training Programme Director, Torbay -NHS England, Yealm Medical Centre, Plymouth, Devon, UK; 2 Devon Partnership NHS Trust, Exeter, England, UK; 3 Health Professions Education and Wellbeing research group, Department of Health and Community Sciences, St Luke’s Campus, University of Exeter Medical School, Exeter, UK; 4 Evidence Synthesis Team, NIHR ARC South West Peninsula (PenARC), St Luke’s Campus, University of Exeter Medical School, Exeter, England, UK

**Keywords:** Clinical Decision-Making, Clinical Reasoning, Education, Medical

## Abstract

**Abstract:**

**Objectives:**

Uncertainty is inherent within medicine. Evidence-based medicine (EBM) teaching within medical curricula seeks to prepare doctors to make clinical decisions within conditions of uncertainty. The goals and scope of training for medical students and doctors are defined by published EBM competencies. We conducted a scoping review to identify the aspects of clinical uncertainty that are addressed by EBM competencies.

**Design:**

A scoping review was conducted to identify and synthesise existing literature and models of clinical uncertainty, with help from an advisory group. Reported according to the Preferred Reporting Items for Systematic Reviews and Meta-Analyses extension for Scoping Reviews.

**Data sources:**

Five databases (MEDLINE, EMBASE, PsycINFO, ERIC and Web of Science Core Collection) and supplementary searches. The searches were performed on 7 March, 2023, repeated and updated on 19 February 2025.

**Eligibility criteria for selecting studies:**

We included empirical research studies that described uncertainty related to clinical decision-making in medical students and doctors.

**Data extraction and synthesis:**

Screening against the inclusion/exclusion criteria, first using titles/abstracts and then full text, was conducted independently by EJ and one other author. Disagreements were discussed and resolved by the full team. Data from included studies were used to create an overarching framework and typology of clinical uncertainty relevant to EBM, which was then mapped to published EBM competencies.

**Results:**

44 of 3048 papers met the inclusion criteria. The overarching framework and typology described types of uncertainty, properties and modifiers of the uncertainty, which led to responses and strategies that clinicians adopted within conditions of uncertainty. The mapping process revealed that EBM competencies focus on the theoretical, technical and cognitive aspects of clinical uncertainty, and tend to neglect real-world clinical decision-making, doctor–patient interaction and behavioural/emotional responses to uncertainty.

**Conclusion:**

The new framework and typology facilitate better conversations about uncertainty within everyday clinical practice. Findings from the mapping exercise can help medical educators to better align EBM teaching with clinical practice, helping them to better prepare doctors for clinical uncertainty. The findings enable different parts of medical curricula to forge stronger connections, for example, linking EBM teaching, communication skills teaching and clinical placements.

**STRENGTHS AND LIMITATIONS OF THIS STUDY**

- The scoping review methodology encompassed the broad sources of literature relevant to uncertainty related to clinical decision-making.
- The broad inclusion criteria, uniquely incorporated reviews and qualitative studies to enhance clinical relevance.
- Experienced evidence-based medicine (EBM) teachers from varied backgrounds supported protocol development, selection of relevant EBM competencies and guided data collation.
- The study was focused on medical students and doctors, rather than wider health groups, which may limit applicability to other health professionals.
- Terminology inconsistencies across publications posed challenges when categorising aspects of uncertainty.

## Introduction

Uncertainty is an inherent part of medicine^1^ with significance for patients and healthcare professionals and the decisions they make.^2^ Therefore, the skills of applying evidence and making decisions in situations of uncertainty are essential to being a doctor, and recognised by medical regulatory bodies internationally.^3–5^ Doctors’ perceptions of uncertainty influence clinical decision-making (CDM), with impacts on their referral rates,^6^ interpretation of test results^7^ and well-being,^8^ and in turn may influence patients’ perception of risk and confidence in their clinician.^9^ Therefore, the effect of uncertainty on how doctors use evidence has significant implications for health service delivery and patient outcomes. This study explores the uncertainty related to CDM and the extent to which education in evidence-based medicine (EBM) is likely to prepare doctors for the realities of practice.

Defining clinical uncertainty remains an ongoing challenge,^10^ not least because of its ‘protean and all-pervasive’ qualities.^11^ While varying definitions for clinical uncertainty exist, for this study it is conceptualised as ‘the subjective perception of ignorance’.^12^ Clinical uncertainty arises from probability ‘the fundamental indeterminacy or randomness of future events’; ambiguity ‘a property of information pertaining to its lack of reliability, credibility or adequacy’ and/or complexity ‘features of a phenomenon that make it difficult to comprehend, eg, multiplicity in its component characteristics, causal determinants or effects’.^12^ An individual’s response to clinical uncertainty can be considered cognitive, emotional or behavioural and these responses can be positive or negative.^13^ New models for clinical uncertainty need to be explored, considered and tailored to the goals of clinical care.^11 14^

EBM was originally defined by Sackett in terms of making decisions about the care of individual patients involving the integration of the best available evidence, with patient values and clinical expertise.^15^ It was conceived as an educational intervention.^16^ It has since become a core part of good clinical practice for doctors and other health professionals, with the terms evidence-based practice and EBM often used interchangeably.^17 18^ The need for doctors to be competent in the skills of EBM is reflected internationally in training competencies for qualified doctors and medical students, for example, the General Medical Council, UK,^19,19 20^or the Accreditation Council for Graduate Medical Education, USA.^21^ Criticism of EBM has focused on where its application does not address clinical uncertainty by losing sight of its original goal, personalising care for individuals^22^ and not addressing probability and complexity,^23^ with ambiguity also being cited by doctors as a barrier for using evidence in their practice.^24^

Despite the ubiquity of uncertainty and its impact on CDM, many medical graduates feel unprepared for dealing with situations involving uncertainty.^25^ EBM training should help to address uncertainties in CDM^17 26^ but its effectiveness in delivering this outcome is unclear.^26^ It appears that the aspects of EBM training that are most likely to address uncertainty may be taught less frequently at medical school^27^ and there is no consistently adopted approach to EBM teaching.^28^ Therefore, core competencies have been described^29^, to form the basis for curricula and learning objectives for teaching.^30^

This study aimed to identify the aspects of clinical uncertainty that are addressed by published EBM competencies. We will explore existing conceptualisations of uncertainty to determine which aspects are relevant to EBM and consider how these aspects are addressed by published EBM competencies.

## Methods

### Study design

A scoping review was conducted to identify the aspects of clinical uncertainty that are addressed by published EBM competencies. The reporting was guided by the Preferred Reporting Items for Systematic Reviews and Meta-Analyses guidelines for scoping reviews,^31^ and the protocol was uploaded to the Open Science Framework.^32^ The scoping review followed the methods described by Arksey and O’Malley ^33^ and developed into the six-step approach (see 1–6 below) by Levac.^34^

#### 1. Identifying the research question

The aim was to identify aspects of clinical uncertainty that are addressed by published competencies in EBM. The research questions were:

1. What aspects of clinical uncertainty are relevant to EBM?
2. Which of these aspects are addressed by published EBM competencies?

#### 2. Identifying relevant studies

The search terms were informed by team discussion and a previous related search.^35^ These terms were piloted with a selection of the title and abstracts from key references and used to create our search strategy (see online supplemental file 1).

CDM was the focus, as it is the conclusion of different components (clinical reasoning, critical thinking, using judgement and gaining understanding of patients)^20^ relevant to the purpose of EBM as originally defined.^17^ In this way, we aimed to identify a broad literature relating to clinical uncertainty, to explore which aspects were relevant to EBM. Both subject headings and free text terms were used, combining terms for models, frameworks or taxonomies with terms for uncertainty and terms for doctors.

MEDLINE, EMBASE and PsycINFO (searched via Ovid), ERIC (via EBSCOhost) and Web of Science Core Collection were searched on 7 March 2023 (online supplemental file 1). The search was repeated and updated on 19 February 2025. Supplementary searches included backward citation chasing of included papers, forward citation chasing using Scopus, a web search using key search terms and an index search of key journals for 2 years (defined as those with three or more included references). Endnote 20 was used to manage the references and for screening. Where papers were not available, the authors were contacted by email to request them. To locate the most recent and applicable EBM competencies, we searched known databases of EBM teaching resources (teachingebhc.org) and Google Scholar, supplemented by backward citation chasing of recent published competencies and input from our advisory group (see later).

#### 3. Selecting studies

The inclusion/exclusion criteria (table 1) were designed to capture a broad literature relevant to clinical uncertainty, while remaining specific and applicable to the context of CDM. Screening against the inclusion/exclusion criteria, first using titles/abstracts and then full text, was conducted independently by EJ and one other author. Disagreements were discussed and resolved by the full team.

**Table 1:** Inclusion and exclusion criteria

	Inclusion	Exclusion
**Sample**	Medical students (undergraduate), trainee doctors, doctors (postgraduate)	Not doctors or medical students (or these representing<50% of the sample)
**Phenomenon of interest**	Uncertainty or ambiguity relating to clinical decision-making	Not relevant to clinical decision-making
**Design**	Research that conceptualises uncertainty (eg, using a theory, model or framework) or presents a thematic analysis (eg, qualitative research, scoping review)	Research that does not add significantly to the conceptualisation of uncertainty
**Research type**	Empirical research of any type (including literature reviews with recognised methodology)	Non-empirical research (eg, editorials, opinion pieces, research without clear methods/data collection)

#### 4. Charting the data

The process of data extraction was iterative. Initial data charting gathered descriptive data about the individual studies and the way in which the papers had defined uncertainty, the origin of the uncertainty and doctors’ responses to this. This informed a second round of data extraction: (1) to specify modifiers of the experience of uncertainty and examples of the responses; (2) to decide on definitions and link related terms (see online supplemental file 2).

#### 5. Collating, summarising and reporting results

The extracted data were collated to identify the aspects of clinical uncertainty relevant to EBM and to link related studies within an **overarching framework**, in order to answer RQ1 (*What aspects of clinical uncertainty are relevant to EBM?*). This provided a discipline and process by which to identify, discuss, clarify and, finally, summarise the different aspects in a ‘**typology’** of clinical uncertainty relevant to EBM. This typology enabled us to answer the second research question (*Which of these aspects are addressed by published EBM competencies?*). To do this, we **mapped** the aspects of uncertainty onto 27 core EBM competencies^29^ and 3 real-world competencies^36^ to determine areas of high and low coverage. There were multiple competencies within each EBM domain. The typology was used to assess how each subtype of uncertainty was addressed by the competency, this was recorded in a matrix (EJ). Within each domain if fewer than a third (1/3) of competencies were addressed, this was deemed low coverage, 1/3–2/3 medium and more than 2/3, high.

#### 6. Consulting with stakeholders and application for the framework to the EBM competencies

Our Stakeholder Advisory Group (see ‘Acknowledgements’) enabled us to consult the intended beneficiaries of our research. The group members were leaders in EBM teaching (through practice and research, and in designing curricula and EBM competencies), from different institutions, geographies and clinical backgrounds. They provided feedback on the study design and data interpretation to ensure that the framework and typology was relevant to EBM teachers. The group advised on current EBM core competencies described in the literature^29^ and stated the importance of a broader ‘meta-cognitive’ approach to EBM, incorporating an understanding of context (eg, when it is appropriate to use pre-appraised evidence), and an attitude of critical thinking. In recognition of this, a real-world EBM competency framework targeting generalist physicians who frequently work in conditions of uncertainty^36^ was used alongside the core competencies (see online supplemental file 3 for each full competency framework.

### Patient and public involvement

None.

## Results

**Figure 1:**
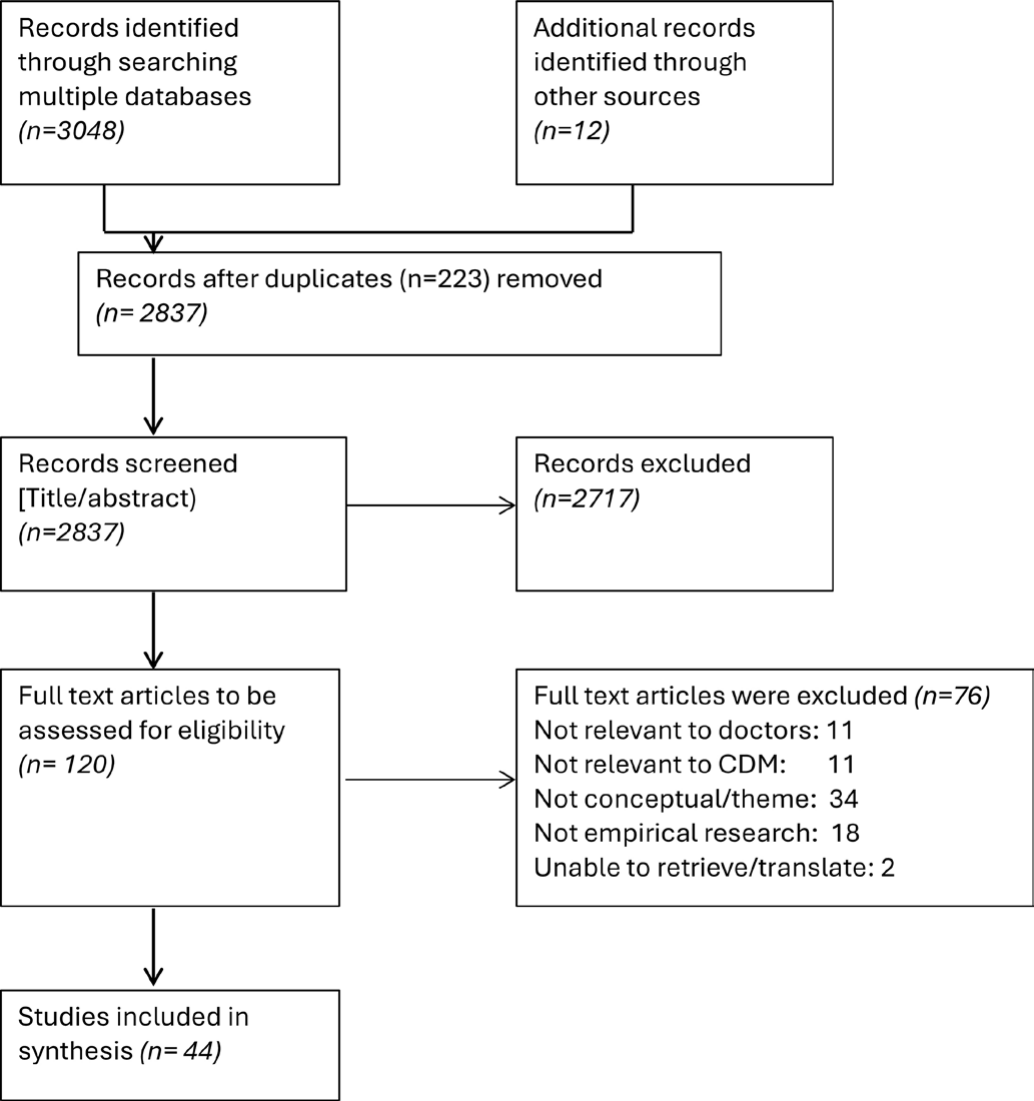
Preferred Reporting Items for Systematic Reviews and Meta-Analyses diagram. CDM, clinical decision-making.

The scoping review identified 2837 papers (after duplicates had been removed), of which 44 met the inclusion criteria (see figure 1). The included studies had diverse study designs: 31/44 were qualitative, 8 were systematic or scoping reviews, 3 were narrative reviews or critical syntheses with conceptual analysis and 2 were mixed methods studies (see table 2). The included studies were from multiple countries: from the USA (18 studies), Australia (4), Europe (4), the UK (3) and Canada (3). The clinical settings were mostly generalist: seven in each of primary care and emergency/general medicine. Most studies involved qualified doctors (33/44) but five involved medical students and six involved both.

**Table 2:** Key characteristics of the 44 included studies

Studies by first author	Aims (summarised)	Study design	Country	Setting/participants
**Adams^61^**	To describe emergency medicine trainees' clinical reasoning using dual cognitive theory	Qualitative	The UK	Emergency medicine, *QD*
**Alam^62^**	To synthesise strategies to manage diagnostic uncertainty, and training that supports them	Systematic review	Multiple	GP, *QD*
**Anderson^63^**	To explore views and reasoning about inappropriate polypharmacy and deprescribing	Qualitative	Australia	GP, pharmacists, *QD*
**Anthony^71^**	To assess uncertainty in CDM, using a framework of message convergence	Qualitative	The USA	Obstetrics, *QD*
**Beresford^37^**	To understand the role of uncertainty in the process of CDM	Qualitative	Canada	Not specified, *QD*
**Belhomme^46^**	To capture the spectrum of uncertainty experiences and build a framework	Qualitative	France	Internal medicine trainees, *QD*
**Bhise^64^**	To synthesise existing literature to define diagnostic uncertainty and its measurement	Systematic review	Multiple	Doctors, *QD*
**Brun^42^**	To assess the influence of intolerance of uncertainty on clinical reasoning.	Scoping review	Multiple	Healthcare workers, *QD*
**Cristancho^47^**	To develop a conceptual framework and a language of uncertainty related to CDM in surgical practice	Qualitative	Canada	Surgery *QD*
**Danczak^65^**	To understand GP trainees’ experience of uncertainty and strategies they use	Qualitative	The UK	GP trainees, *QD*
**Diamond-** **Brown^72^**	To consider responses to uncertainty using doctor–patient relationship as toolkit.	Qualitative	The USA	Obstetrics, *QD*
**Djulbegovic^66^**	To assess physicians’ decision-making with reference to a threshold model	Mixed methods	The USA	Doctors *QD*
**Eastwood^43^**	To assess what is known about epistemic cognition in medical education	Systematic review	The USA	Medical school, *MS*
**Ely^56^**	To describe the obstacles when attempting to answer doctors’ questions with evidence	Qualitative	The USA	GP, *QD*
**Farnan^38^**	To describe critical incidents occurring as a result of uncertainty, its origin and related CDM strategies	Qualitative	The USA	General medicine trainees, *QD*
**Gartner^73^**	To explore language used to implicitly express uncertainty	Qualitative	Germany	Medical school, *MS*
**Griffiths^57^**	To describe how doctors deal with the uncertainty in medical evidence in clinical consultations	Qualitative	The UK	GP, breast surgery, *QD*
**Hamui-Sutton** ^39^	To identify the relation between types of uncertainty and coping strategies, build on Beresford’s typology	Mixed methods	Mexico	General medicine trainees, *QD*
**Han (**1)^9^	To summarise major theoretical insights relevant to understanding uncertainty specific to healthcare and to develop an integrative conceptual taxonomy	Narrative review /analysis	Multiple	Healthcare professionals, *QD /MS*
**Han (**2)^48^	To explore the uncertainty management strategies and organise these within a conceptual taxonomy	Qualitative	The USA	General medicine, *QD*
**Helou^67^**	To explore uncertainty in CDM across disciplines to formulate a model describing the role of uncertainty in medicine in the decision-making process	Scoping review/ analysis	Multiple	>50% health sciences literature, *QD/MS*
**Hewson^68^**	To identify strategies involved in diagnosis and making treatment plans for uncertain and complex problems	Qualitative	The USA	GP, general medicine, *QD*
**Hill^74^**	To describe the meaning of uncertainty regarding paediatric oncologists making palliative care referrals	Qualitative	The USA	Paediatric oncology, *QD*
**Hillen^13^**	To analyse the meaning and logical coherence of UT as described by UT measures to develop an integrative conceptual model	Narrative review /analysis	The USA	Multiple, *QD/MS*
**Ilgen (**1)^50^	To explore what it means to be ‘comfortable’ when working in clinical situations while feeling ‘uncertain’	Critical synthesis	Multiple	Multiple, *QD*
**Ilgen (**2)^49^	To explore physicians’ experiences with uncertainty and how they assess when they can capably handle uncertain situations safely and effectively	Qualitative	The USA	Emergency medicine, *QD*
**Ilgen (**3)^51^	To explore clinicians’ responses to discomfort in settings of uncertainty and how they still provided effective care	Qualitative	The USA	Emergency medicine, *QD*
**Ilgen (**4)^52^	To explore trainees’ experiences of uncertainty, their level of comfort and their responses	Qualitative	The USA	Emergency medicine trainees, *QD*
**Islam^69^**	To examine CDM and explore the cognitive strategies used to control and adapt to their information environment	Qualitative	The USA	Infectious diseases, *QD*
**Ivany^58^**	To explore CDM regarding stroke prevention in patients with atrial fibrillation and a recent intracerebral bleed	Qualitative	Europe	Stroke medicine, *QD*
**Jones^75^**	To identify strategies used to communicate uncertainty in the context of CDM for patients with traumatic brain injury	Qualitative	The USA	Emergency medicine, *QD*
**Kerr^76^**	To address what is known about paediatricians’ goals and their effect, when discussing uncertainty	Qualitative	The USA	Paediatrics, *QD*
**Lee^14^**	To explore existing models of clinical uncertainty and develop a framework to aid medical education	Scoping review /analysis	Multiple	Doctors, *QD/MS*
**Lingard^77^**	To describe the rhetorical and linguistic features of uncertainty in case presentation	Qualitative	Canada	Paediatrics, *QD/MS*
**Loewenthal^53^**	To understand the challenges re. caring for older patients through the concepts of uncertainty and moral distress	Qualitative	The USA	Elderly care trainees, *QD*
**Moffett^44^**	To explore how students learn to engage with uncertainty related to their academic practice	Scoping review	Multiple	Medical school, *MS*
**Mutter^54^**	To explore sources of, approaches to and feelings about uncertainty in medical decision-making	Qualitative	The USA	General medicine trainees, *QD*
**Patel^35^**	To study the cognitive, communication and management behaviours regarding diagnostic uncertainty	Qualitative	The USA	Paediatrics, *QD*
**Pickles^40^**	To explore the sources of uncertainty as described by GPs and how they experience and respond to these in the context of prostate-specific antigen testing	Qualitative	Australia	GP, *QD*
**Pomare^41^**	To examine the applicability of the existing range of issues delineated in a taxonomy of uncertainty (Beresford) in different settings, and how the issues inter-relate	Scoping review	Multiple	Healthcare professionals, *QD/MS*
**Richter^59^**	To explore the risk communication strategies used by clinicians to help inform medical education	Qualitative	The Netherlands	Doctors, *QD*
**Stephens (**1)^45^	To identify the impacts of education on tolerance of uncertainty in the context of a core medical curriculum	Qualitative	Australia	Medical school, *MS*
**Stephens (**2)^55^	To describe how medical students respond to uncertainty, how this changes over time and contributes to understanding UT	Qualitative	Australia	Medical school, *MS*
**Timmermans^60^**	To evaluate the extent to which the medical sociology scholarship on uncertainty, analytically elucidates the impact of EBM training during residency	Qualitative	Australia	Paediatrics, *QD*

CDM, clinical decision-making; EBM, evidence-based medicine; GP, general practice; MS, medical students; QD, qualified doctors; UT, uncertainty tolerance.

### Q1: What aspects of clinical uncertainty are relevant to EBM?

Six groups of studies were identified based on the way they described uncertainty relevant to EBM (see online supplemental file 4):

1. 8 studies set out or built on conceptual frameworks^13 12 37 38 39 40 41 14^
2. 4 studies described external factors affecting experience.^42–45^
3. 10 studies described ‘in-the-moment’ experience.^46–55^
4. 5 studies described how information was managed.^56–60^
5. 10 studies described clinical decision-making actions.^61–70^
6. 7 studies described the communication of uncertainty.^71–77^

No single identified model or framework was sufficient to address all aspects of uncertainty that may relate to EBM.

**Figure 2:**
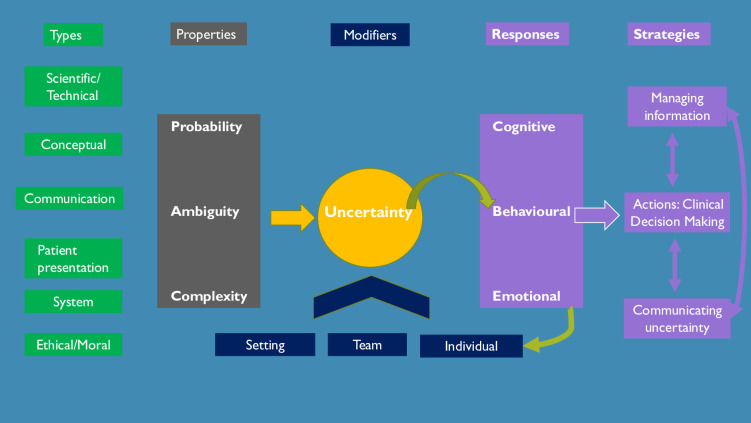
The Overarching framework of Clinical Uncertainty as it Relates to Evidence Based Medicine. The framework describes how the aspects of uncertainty relate, key linkages are shown by arrows. The six groups of studies that underpin the framework are recognisable in its structure: papers in the first group (studies that set out conceptual frameworks) informed the overall structure. Papers in the second group (describing external factors affecting the experience) are evident in the ‘types’ of uncertainty. Other groups are identifiable in the ‘strategies’ on the right hand side.

Therefore, the six groups of studies were collated into an **overarching framework** (figure 2), which provided specific, new insights into the different aspects of uncertainty relevant to EBM described by the included papers and how they interrelated. Qualitative studies of clinical practice were particularly helpful for identifying different aspects of uncertainty and their relationships.

The framework alone was insufficient for answering RQ2. It provided an overview but did not provide the required level of detail in the definitions of the aspects of uncertainty necessary to analyse these against the EBM competencies. Thus, we then created **a typology** of clinical uncertainty applicable to EBM see (online supplemental files 5 and 6). The different aspects were further delineated into ‘types’ of uncertainty, ‘properties’ of the uncertainty (eg, probability, ambiguity, complexity) and ‘modifiers’ of the uncertainty, ‘responses’ to the uncertainty, for example (cognitive, behavioural, emotional) and clinician strategies adopted (eg, for information management, CDM and communicating uncertainty).

The aspects defined in the typology were either derived from existing models (eg, probability, ambiguity, complexity from the Han model) or were new, arising from the synthesis process (eg, patient presentation).

### Q2: Which of the aspects of uncertainty are addressed by published EBM competencies?

Table 3 mapped aspects of uncertainty (defined by the typology) to the 30 competencies (27 core EBM competencies grouped into steps 0–5 and 3 real-world competencies). Some aspects of uncertainty were addressed well by published EBM competencies. For example, the ‘scientific/technical’ and ‘conceptual’ types of uncertainty were widely addressed by steps 1–3 of the core EBM competencies. The ‘ambiguity’ property of uncertainty and the cognitive response to uncertainty were both broadly addressed.

**Table 3:** Summary matrix showing the aspects of uncertainty (in the rows) mapped to evidence-based medicine core and real-world published competencies (in the columns)

Aspects of uncertainty/EBM Competencies	0 Introduction	1 Ask	2 Acquire	3 Interpret appraise	4 Apply	5 Evaluate	Real-world (m/p/k)
Type							
Scientific/technical							
Conceptual							
Patient presentation							
Personal/communication							
Practical/system							
Ethical/moral							
Properties							
Ambiguity							
Probability							
Complexity							
Modifiers							
Demographic							
System							
Doctor characteristics							
Response: dimension							
Cognitive							
Behavioural							
Emotional							
Response: actions							
Managing evidence							
CDM strategies							
Communicating uncertainty							

The shaded cells in the matrix reflect where the aspect of uncertainty was addressed by teaching competencies within each step of evidence-based medicine: white: 0–1/3 of competencies, light grey: 1/3–2/3 of competencies, dark grey: >2/3 of competencies.

Real-world competencies: m, mindfulness; p, practical; k, knowledge.

CDM, clinical decision-making.

Other aspects were addressed in a limited way by the competencies. These were patient presentation, practical/system, ethical types of uncertainty and the properties of uncertainty relating to probability and ‘complexity’. These were addressed by 11/30 and 7/30 competencies, respectively. Some aspects were almost exclusively addressed by the step 4, 5 or ‘real-world’ competencies. These were the modifiers of uncertainty, and the behavioural/emotional response, which included communicating uncertainty and CDM strategies.

## Discussion

This study identified the aspects of clinical uncertainty that are addressed by published EBM competencies. This was achieved by creating a framework and typology for understanding clinical uncertainty that can be applied to EBM competencies. The aspects in the framework were then mapped against established ‘core’ EBM competencies and a broader set of ‘real-world’ competencies. The use of the framework to map coverage of EBM competencies is novel and the approach may be transferrable to other clinical reasoning processes such as shared decision-making.^78^ Our study provides helpful insights for clinical practice and demonstrates how EBM teaching can be designed to better support this. It has implications for the debate about the future composition of EBM and its related training.^79^

The framework draws together established models^12 37^ with more recent concepts^13 50^ and considers how the aspects link. Despite arising from a different approach (scoping review), the framework is similar to Han and Hillen’s models,^12 13^ in describing uncertainty in terms of its origin, modifiers and response. It also extends this with a broader range of aspects of uncertainty described and details on how they link. Six types of uncertainty were described: scientific/technical; communication/personal; conceptual; practical/systemic; moral/ethical and patient presentations. ‘Patient presentation’ has not previously been described as an aspect of uncertainty previously and arose from qualitative studies defining incongruent or discordant information from patients as a cause of uncertainty.^76^ The framework shows the practitioner and the context in which they work as dynamic parts of a complex system which affect how a moment of uncertainty is experienced. In turn, the clinicians’ responses described feedback into the system. These modifiers and responses to the aspects of uncertainty were informed by work observing clinical practice^46–49 51–53 55^

The mapping identified the aspects of uncertainty that are and are not addressed by EBM competencies. The findings suggest that EBM teaching is focussing on the theoretical, technical and cognitive aspects, although with an appreciation of ambiguity, and a reasonable focus on personal communication. Notable aspects addressed in a limited way were the personal/communication type of uncertainty, the properties of probability and complexity, and the responses of communicating uncertainty or using broader CDM strategies. This suggests that EBM teaching is not focussing on real-world CDM, the doctor–patient interaction or the behavioural/emotional responses of the doctor to uncertainty in clinical practice.

Using uncertainty as a lens through which to examine EBM is relevant to current issues and debates regarding the need for new approaches for EBM teaching, such as how to translate evidence (about groups) to treatment decisions at an individual level, and how to share and communicate the probability and complexity involved.^22 26^ It is of note that many of these criticisms relate to the areas of low coverage identified.

### Implications

#### For clinical practice

The typology provides doctors with a set of terms to describe uncertainty, thus addressing the need to agree on common definitions in the field^10^ while acknowledging the likely impossibility of an all-encompassing model.^11^ The framework is important as doctors often experience uncertainty while applying evidence in the complex reality of clinical practice but feel unprepared for this.^25^ Recognising and naming aspects of uncertainty helps clinicians to challenge the feeling that uncertainty is to be feared and avoided.^80^ It also enables doctors to consider their response to this feeling, and that can be adaptive or maladaptive.^55^ The typology can help doctors with effective communication about uncertainty to each other and their patients. More effective approaches to defining and communicating uncertainty have been identified as necessary to explore the extent or otherwise of evidence and to advance shared decision-making. This paper addresses this need, and in so doing has the potential to improve how clinicians apply population-based evidence with their individual patients.^81^

The framework can be used to describe how doctors use evidence in situations of clinical uncertainty and relate these to different CDM strategies. It highlights the different skills and support required, and how these are influenced by the clinical context. In acute settings like emergency departments (EDs), with high levels of diagnostic uncertainty, an iterative approach is used with ED doctors using strategies such as watchful waiting to manage ambiguity and complexity.^52^ This contrasts with outpatient clinics, where decisions typically focus on longer-term care, for example, how to manage cardiovascular risk or whether a patient should participate in breast cancer screening.^57 58^ In these scenarios, doctors used shared decision-making (step 4 of EBM) as a strategy for managing uncertainty.

This has implications for the design of specific clinical systems that best support doctors’ CDM. In ED, consideration is needed of what resources, material or human that clinicians need in the moment.^52^ Whereas in outpatient clinic settings, access to infographics and evidence summaries should be prioritised to support doctors communicating probabilistic uncertainty.

#### For medical education

The application of evidence with an individual patient (step 4 of EBM) requires competencies that address both scientific/technical and communication types at the same time. It also means training doctors to address probabilistic uncertainty. The mapping shows this is under-represented in the current core competencies. EBM and shared decision-making have often been taught separately.^82 83^ Connecting these skills together and building on existing tools^29 84^ will enable EBM teaching to better relate to uncertainty from communication and probability.

Applying evidence in complex, uncertain situations is recognised as a core competency for doctors.^85^ Although educational models addressing this exist,^86^ they have not been widely incorporated into EBM training. The mapping process shows how these skills could be developed by doctors by incorporating the broader competencies (step 5/‘real-world’) into EBM training. Specifically, these competencies set out to develop broader, metacognitive skills in doctors: self-awareness and pragmatism. Self-awareness is an important component of expertise; it enables uncertainty to be recognised and judgement used as to how to apply evidence to that individual.^87^ Pragmatism is used to recognise the system constraints in which you work in order to address practical/system-type uncertainties. Incorporating this competency would encourage medical education to make explicit the impact broader factors in health systems (such as limited resources) have on applying EBM. Addressing these sources of uncertainty as part of EBM teaching would enhance its clinical relevance and potentially its effectiveness.^88^

There is work to be done in considering how to operationalise the tacit, real-world competencies, which may involve on the job rather than didactic methods of teaching. The competencies of EBM are a proxy for what EBM is in practice or in training; the typology and framework could inform ethnographic or qualitative studies to examine this.

### Strengths and weaknesses

A scoping review was an appropriate methodology since uncertainty has been considered through medical, psychological and social lenses^12^ with diverse study designs. This is reflected in the breadth of literature searched in our study. The inclusion criteria were also broad and allowed the inclusion of reviews and qualitative studies. This is the first review in this field to do this and helps add clinical context.

The stakeholder group were experienced EBM teachers from diverse backgrounds. They helped with protocol design, ensuring the relevant EBM competencies were selected and evaluating the results.

The review included references regarding clinical uncertainty in doctors and medical students rather than in wider healthcare professionals or patients. This was because CDM has mostly been described in doctors and this aligned with our expertise. We recognise this may limit the applicability of the findings. Judgements had to be made about the inclusion of qualitative studies regarding novelty of contribution, which introduces an element of subjectivity. The process of mapping the aspects of uncertainty to the competencies was done by one author, with expertise in EBM teaching and the competencies had several clauses which had to be combined.

Another limitation was the challenges of terminology in this area of research. Not only was there the challenge of the specific definitions of uncertainty, ambiguity, EBM, etc., but determining appropriate terminology for the different aspects of uncertainty described by the typology, as different included publications used different terms ().

## Conclusion

The new framework and typology may facilitate better conversations about uncertainty within everyday clinical practice. Findings from the mapping exercise can help medical educators to better align EBM teaching with clinical practice, helping them to better prepare doctors for clinical uncertainty. The findings may also enable different parts of medical curricula to forge stronger connections, for example, linking EBM teaching, communication skills teaching and clinical placements.

## Supplementary material

10.1136/bmjopen-2025-109013 online supplemental file 1

10.1136/bmjopen-2025-109013 online supplemental file 2

10.1136/bmjopen-2025-109013 online supplemental file 3

10.1136/bmjopen-2025-109013 online supplemental file 4

10.1136/bmjopen-2025-109013 online supplemental file 5

10.1136/bmjopen-2025-109013 online supplemental file 6

## Data Availability

All data relevant to the study are included in the article or uploaded as supplementary information.
