# Supplementary material for: What aspects of clinical uncertainty are addressed by published competencies in evidence-based medicine? A scoping review
Source: BMJ Open. 2026 Jul 31;16(7):e109013. doi: 10.1136/bmjopen-2025-109013 (PMC13435916; doi:10.1136/bmjopen-2025-109013)
Supplement: Supplementary file 1 [file bmjopen-16-7-s001.pdf]

Embase, Search 7/3/23		
	**Search Query**	**Results**
1	Educational model/	8392
2	(model/ or *information model/ or *theoretical model/) and model.ti.	76787
3	framework*.ti,ab	424110
4	taxonom*.ti,ab.	82535
5	theory/ or conceptual framework/ or hypothesis/ or systems theory/	213014
6	thematic analysis/ or concept analysis/ or qualitative analysis/	111242
7	phenomenen*.ti,ab.	19
8	1 or 2 or 3 or 4 or 5 or 6 or 7	543219
9	uncertainty/	49570
10	uncertainty.ti,ab.	122062
11	*ambiguity/	622
12	ambiguity.ti,ab.	16881
13	9 or 10 or 11 or 12	151609
14	physician*.ti,ab.	645156
15	exp physician/	952231
16	resident/	70016
17	doctor*.ti,ab.	212768
18	resident*.ti,ab.	303940
19	medical student.ti,ab.	14841
20	exp medical student/	86948
21	14 or 15 or 16 or 17 or 18 or 19 or 20	1752947
22	8 and 13 and 21	1732

Medline, Search 7/3/23		
1	*Models, Theoretical/	64535
2	(model/ or *information model/ or *theoretical model/) and model.ti.	13664
3	*models, educational/	3965
4	framework*.ti,ab.	383027
5	taxonom*.ti,ab.	81363
6	theory.ti,ab.	409386
7	phenomenen.ti,ab.	3
8	1 or 2 or 3 or 4 or 5 or 6 or 7	891100
9	*uncertainty/	4857
10	uncertainty.ti,ab.	97509
11	ambiguity.ti,ab.	14450
12	9 or 10 or 11	111991
13	*Physicians/	73613
14	physician*.ti,ab.	445672
15	doctor*.ti,ab.	148129
16	*"Internship and Residency"/	43897
17	*Students, Medical/	32004
18	medical student.ti,ab.	9072
19	resident.ti,ab.	82972
20	13 or 14 or 15 or 16 or 17 or 18 or 19	720378
21	8 and 12 and 20	682

APA PsycInfo [Using Medline terms], Search 7/3/2023		
1	*Models, Theoretical/	0
2	(model/ or *information model/ or *theoretical model/) and model.ti.	0
3	*models, educational/	0
4	framework*.ti,ab.	229646
5	taxonom*.ti,ab.	11704
6	theory.ti,ab.	412462
7	phenomenen.ti,ab.	0
8	1 or 2 or 3 or 4 or 5 or 6 or 7	597647
9	*uncertainty/	7867
10	uncertainty.ti,ab.	34855
11	ambiguity.ti,ab.	14946
12	9 or 10 or 11	49594
13	*Physicians/	15640
14	physician*.ti,ab.	68203
15	doctor*.ti,ab.	43353
16	*"Internship and Residency"/	0
17	*Students, Medical/	0
18	medical student.ti,ab.	2366
19	resident.ti,ab.	14311
20	13 or 14 or 15 or 16 or 17 or 18 or 19	121408
21	8 and 12 and 20	239

ERIC Database, Search 7/3/23		
S1	DE "Educational Theories" OR DE "Taxonomy"	13,337
S2	TI (framework or model or theory) OR AB (framework or model or theory)	371,765
S3	TI (phenomenology or phenomenological or lived experience) OR AB (phenomenology or phenomenological or lived experience)	12,148
S4	TI uncertainty OR AB uncertainty	6,399
S5	DE "Ambiguity (Context)" OR TI ambiguity OR AB ambiguity	4,332
S6	(DE "Physicians") OR (DE "Medical Students")	8,750
S7	TI physician* OR AB Physician*	5,608
S8	TI doctor* OR AB doctor*	16,021
S9	S1 OR S2 OR S3	384,854
S10	S4 OR S5	10,439
S11	S6 OR S7 OR S8	26,463
S12	S9 AND S10 AND S11	73

Web of Science, Database Search 7/3/23		
1	uncertainty (Topic)	642652
2	ambiguity (Topic)	83827
3	physician* (Topic)	398480
4	doctor* (Topic)	170730
5	medical student (Topic)	95250
6	#1 OR #2	717903
7	#3 OR #4 OR #5	618539
8	Framework (Abstract)	1496458
9	taxonomy (Title)	23863
10	taxonomy (Abstract)	56569
11	phenomenon (Title)	98459
12	phenomenon (Abstract)	892735
13	theory (Abstract)	1990502
14	theory (Title)	624570
15	framework (Title)	262194
16	model (Title)	2825281
17	#8 OR #9 OR #10 OR #11 OR #12 OR #13 OR #14 OR #15 OR #16	7124256
18	#17 AND #7 AND #6	1350

Supplementary Material Figure 3: search strategies for different databases
