# Supplementary material for: What aspects of clinical uncertainty are addressed by published competencies in evidence-based medicine? A scoping review
Source: BMJ Open. 2026 Jul 31;16(7):e109013. doi: 10.1136/bmjopen-2025-109013 (PMC13435916; doi:10.1136/bmjopen-2025-109013)
Supplement: Supplementary file 2 [file bmjopen-16-7-s002.pdf]

Aspect of Uncertainty	Terms used		Selected term for framework	Definition from OED unless stated
**Origin**	Type (Beres-ford)	Issue (Han i)	**Type:**	a category of people or things having common characteristics.
	Source (Han i)	Property (Ilgen i)	**Property:**	An attribute, quality, or characteristic of something<br>(for uncertainty: probability, ambiguity, complexity)
**Modifier**			**Modifier:**	A thing that makes.. partial changes to something
**Response**			Response: **Domain**	Refers to how doctors think (cognitive), act (behavioural), feel (emotional) in response to uncertainty (Lee)
			Response: **Action**	The.. process of doing something, typically to achieve an aim.

Supplemental Material Figure 4: Defining terminology related to Uncertainty:

A review of the terminology used to refer to different aspects of uncertainty was carried out. This was done with the aim of clarifying and simplifying terms used to describe the aspects of uncertainty.

It was performed during the 2nd round of data extraction and used The Oxford English Dictionary (OED) The figure describes where the various terms used to describe uncertainty originate, how they relate and the combined terms we adopted.
