# Supplementary material for: What aspects of clinical uncertainty are addressed by published competencies in evidence-based medicine? A scoping review
Source: BMJ Open. 2026 Jul 31;16(7):e109013. doi: 10.1136/bmjopen-2025-109013 (PMC13435916; doi:10.1136/bmjopen-2025-109013)
Supplement: Supplementary file 3 [file bmjopen-16-7-s003.pdf]

## Set of EBP Core Competencies Grouped Into the Main EBP Domains

| EBP Core Competency | Rating |
|---|---|
| **0. Introductory** | |
| 0.1 Understand EBP defined as integration of best research evidence with clinical expertise and patient's unique values and circumstances* | E |
| 0.2 Recognise the rationale for EBP | M |
| *The daily clinical need for valid information to inform decision making, and the inadequacy of traditional sources* | |
| *The disparity between diagnostic skills and clinical judgment, which increase with experience, and up-to-date knowledge and performance, which decline with age* | |
| *Lack of time to find and assimilate evidence as a clinician* | |
| *The gaps between evidence and practice can lead to suboptimal practice and quality of care* | |
| *The potential discordance between a pathophysiological and empirical approach to thinking about effectiveness** | |
| 0.3 For each type of clinical question, identify the preferred order of study designs, including pros and cons of the major study designs* | E |
| *Classify the major study designs for each type of clinical question* | |
| 0.4 Practise the 5 steps of EBP: ask, acquire, appraise, apply, and evaluate* | P |
| 0.5 Understand the distinction between using research to inform clinical decision making vs conducting research* | M |
| **1. Ask** | |
| 1.1 Explain the difference between background questions (cannot typically be answered by research) and foreground questions* | E |
| 1.2 Identify different types of clinical questions: treatment, diagnosis, prognosis, and etiology* | P |
| 1.3 Convert clinical questions into structured, answerable clinical questions using PICO* | P |
| *Recognise the importance of and strategies for identifying and prioritising uncertainties or knowledge gaps in practice* | |
| *Understand the rationale for using structured clinical questions* | |
| *Identify PICO elements and use variations when appropriate (e.g. PICOT, PO, PECO–Exposure)** | |
| **2. Acquire** | |
| 2.1 Outline the different major categories of sources of research information, including biomedical databases and filtered/preappraised resources* | E |
| *Outline the advantages of filtered or preappraised evidence sources and recognise relevant resources* | |
| *Indicate the differences between hierarchy of evidence, level of processing, and types of EBM resources** | |
| 2.2 Construct and carry out an appropriate search strategy for clinical questions* | P |
| *Know where to look first to address a clinical question* | |
| *When necessary, construct a search strategy that reflects the purpose of the search** | |
| *Apply a general search strategy including use of search terms, Boolean operators, truncation, and search filters** | |
| 2.3 State the differences in broad topics covered by the major research databases | M |
| 2.4 Outline strategies to obtain the full text of articles and other evidence resources* | E |
| **3. Appraise and Interpret** | |
| 3.1 Identify key competencies relevant to critical evaluation of the integrity, reliability, and applicability of health-related research | E |
| *Understand the difference between random error and systematic error (bias)** | |
| *Identify the major categories of bias and the impact of these biases on results** | |
| *Interpret commonly used measures of uncertainty, in particular confidence intervals** | |
| *Recognise that association does not imply causation and explain why** | |
| *Recognise the importance of considering conflict of interest and funding sources* | |
| *Recognise the uses and limitations of subgroup analysis and how to interpret results** | |
| 3.2 Interpret different types of measures of association and effect, including key graphical presentations* | P |
| *Identify the basic types of data such as categorical and continuous** | |
| *Recognise the meaning of some basic frequency measures* | |
| *Identify the difference between 'statistical significance' and 'importance,' and between lack of evidence of an effect and 'evidence of no effect'** | |
| 3.3 Critically appraise and interpret a systematic review* | P |
| *Recognise the difference between systematic reviews, meta-analyses, and nonsystematic reviews** | |
| *Identify and critically appraise key elements of a systematic review* | |
| *Interpret presentations of pooling of studies such as a forest plot and summary of findings table* | |
| 3.4 Critically appraise and interpret a treatment study* | P |
| *Identify and appraise key features of a controlled trial* | |

| EBP Core Competency | Rating |
|---|---|
| *Interpret the results, including measures of effect* | |
| *Identify the limitations of observational studies as treatment studies, and recognise the basics of adjustment methods and their limitations* | |
| 3.5 Critically appraise and interpret a diagnostic accuracy study* | P |
| *Identify and appraise key features of a diagnostic accuracy study* | |
| *Interpret the results, including measures to evaluate diagnostic accuracy** | |
| *Recognise the purpose and use of clinical prediction rules* | |
| 3.6 Distinguish evidence-based from opinion-based clinical practice guidelines* | P |
| 3.7 Identify the key features of, and be able to interpret, a prognostic study | E |
| *Identify and appraise key features of a prognostic study* | |
| *Interpret results including measures of effect (e.g. Kaplan-Meier survival curves) and uncertainty* | |
| *Recognise the purpose and use of clinical prediction rules* | |
| 3.8 Explain the use of harm and etiologies study for (rare) adverse effects of interventions* | E |
| *Indicate that common treatment harms can usually be observed in controlled trials, but rare or late harms will only be seen in observational studies* | |
| 3.9 Explain the purpose and nature of a qualitative study | E |
| *Recognise how qualitative research can inform the decision making process* | |
| **4. Apply** | |
| 4.1 Engage patients in the decision making process using shared decision making, including explaining evidence and integrating their preferences* | P |
| *Recognise the nature of the patient's dilemma, hopes, expectations, fears, and values and preferences* | |
| *Understand and practise shared decision making* | |
| *Recognise how decision support tools such as patient decision aids can assist in shared decision making* | |
| 4.2 Outline different strategies to manage uncertainty in clinical decision making in practice | E |
| *Recognise professional, ethical, and legal components and dimensions of clinical decision making, and the role of clinical reasoning* | |
| 4.3 Explain the importance of baseline risk of individual patients when estimating individual expected benefit | E |
| *Recognise different types of outcome measures (surrogate vs composite endpoints)* | |
| 4.4 Interpret the grading of the certainty in evidence and the strength of recommendations in health care | E |
| **5. Evaluate** | |
| 5.1 Recognise potential individual-level barriers to knowledge translation and strategies to overcome these | M |
| *Recognise the process of reflective clinical practice* | |
| 5.2 Recognise the role of personal clinical audit in facilitating EBP | M |

**Abbreviations:** E, explained; EBP, evidence-based practice; M, mentioned; P, practised with exercises; PECO, patient, exposure, comparison, outcome; PICO, patient, intervention, comparison, outcome; PICOT, population, intervention, comparison, outcome, time; PO, population, outcome. ** Core competencies that achieved consensus level in Delphi round 2.*

**Source: Albarqouni L, Hoffman T, Strauss S, et al. Core Competencies in Evidence-Based Practice for Health Professionals: Consensus Statement Based on a Systematic Review and Delphi Survey. JAMA Netw. Open.2018; 1(2):e180281. Doi:10.1001/jamanetwrokopen.2018.0281**

| Real-World EBM Competency | Domain |
|---|---|
| **1.1 Mindfulness in one's approach toward EBM itself,** and approaches to the influences on clinical decision-making (CDM)<br>*Awareness of cognitive biases and heuristics that affect clinical reasoning*<br>*Recognition of the role of values, experience, and context in shaping decisions*<br>*Reflective practice regarding one's own use of evidence in clinical settings* | **Mindfulness** |
| **2.1 Pragmatism in one's approach** to finding and evaluating evidence<br>*Ability to identify and access the most useful evidence efficiently in a real clinical context*<br>*Critically evaluate evidence in a time-efficient manner appropriate to the clinical setting*<br>*Balance the ideal of best available evidence with practical constraints of clinical practice* | **Pragmatism** |
| **3.1 Knowledge of the patient** as the most useful resource in effective evidence-based CDM<br>*Integrate detailed knowledge of the individual patient's circumstances, values, and preferences* | **Patient Knowledge** |

| | |
|---|---|
| *Recognise that patient context is central to applying evidence appropriately*<br>*Use the therapeutic relationship as a source of evidence alongside research findings* | |

***Source: Galbraith K, Ward A, Heneghan C. A real-world approach to Evidence-Based Medicine in general practice: a competency framework derived from a systematic review and Delphi process. BMC Medical Education. 2017 Dec;17(1):1–5.***

**Supplemental Material Figure 5: Full set of EBM Competencies, Core and Real-world GP**
