# Supplementary material for: What aspects of clinical uncertainty are addressed by published competencies in evidence-based medicine? A scoping review
Source: BMJ Open. 2026 Jul 31;16(7):e109013. doi: 10.1136/bmjopen-2025-109013 (PMC13435916; doi:10.1136/bmjopen-2025-109013)
Supplement: Supplementary file 4 [file bmjopen-16-7-s004.pdf]

Group	Name	No. of studies	Included Studies	Origin		Modifiers	Responses	Key Features
				Types	Property			
**1**	Overarching conceptual frameworks	8/44	Beresford, Farnan, Hamui-Sutton. Han (i), Pickles, Pomare, Hillen, Lee.	++	+<br>+	++	+<br>+	• Beresford originally described types: technical, conceptual, personal. Types added: ethical/system and communication (replaces personal)<br>• Han defines types: scientific, practical, personal; also complexity, ambiguity, probability (properties – see appendix figure 4)<br>• Hillen/Lee describe modifiers and response
**2**	External factors affecting experience	4/44	Brun, Eastwood, Moffett, Stephens.	+	-	++	+	• Types, related to education/learning.<br>• Modifiers: typical external eg. age/gender/accrued experience<br>• Response: broadly described as cognitive/behavioural/emotional and negative
**3**	'In-the-moment' experience	10/44	Belhomme, Cristancho, Han (ii), Ilgen (i-iv) Lowenethal Mutter, Stephens ii	+	+	++	+<br>+	• In the moment experience, 'What's going on?' 'What should I do?'<br>• Modified by context (E.D./surgery) and patient<br>• Responses: increased cognitive attention, gathering resources, recognising gut feeling/lack of fluency<br>• Emotional response described as forms of distress but can be a trigger for adaptive or maladaptive behaviours

**4**	Managing the evidence	5/44	Ely, Ivany, Timmermans, Griffiths, Richter	+	+	-	+ +	• Triggered by lack or conflicting evidence about what to do for a patient<br>• Responses described how to find, use or explain evidence to their patients (including SDM)
**5**	Clinical decision making strategies	10/44	Adams, Anderson, Danczak, Djulbegovic, Helou, Hewson, Islan, Patel, Alan, Bhise	+	-	+	+ +	• Recognition of uncertainty, type not specified.<br>• Described specific strategies in response including:<br>- Restricted rule out; Pause and review: Using a working diagnosis (Iterative); Safety netting; discussing with colleagues; SDM; Probabilistic reasoning; tests of time/treatment
**6**	Communicating uncertainty itself	7/44	Anthony, Diamond-Brown, Jones, Kerr, Lingard, Gartner, Hill.	++	+	-	+ +	• Type, communication with patients and colleagues could cause uncertainty, eg. not fitting illness script or lack of information<br>• Response: identified strategies and language used to communicate uncertainty itself

Supplemental Material Table 5: Groups collated by common features in the extracted data
Key: ED – Emergency Department, SDM: Shared decision making.

- *Little to no mention; + mention, ++ focus of studies*
