# Supplementary material for: What aspects of clinical uncertainty are addressed by published competencies in evidence-based medicine? A scoping review
Source: BMJ Open. 2026 Jul 31;16(7):e109013. doi: 10.1136/bmjopen-2025-109013 (PMC13435916; doi:10.1136/bmjopen-2025-109013)
Supplement: Supplementary file 5 [file bmjopen-16-7-s005.pdf]

Type	Sub-type	Adams	Alam	Anderson	Anthony	Belhomme	Beresford	Bhise	Brun	Cristancho	Danczak	Diamond-B	Djulbegovic	Eastwood	Ely	Farnan	Gartner	Griffiths	Hamui-S	Han 11	Han 21	Helou	Hewson	Hill	Hillen	Ilgen [i]	Ilgen [ii]	Ilgen [iii]	Ilgen [iv]	Islan	Ivany	Jones	Kerr	Lee	Lingard	Loewenthal	Moffett	Mutter	Patel	Pickles	Pomare	Richter	Stephens (i)	Stephens (ii)	Timmermans
Scientific/ Technical	Treatment: Evidence			x	x	x	x					x						x	x	x		x	x				x			x	x			x		x		x		x	x	x			x
	Treatment: Procedural					x				x						x											x																		
	Diagnostic	x	x			x		x								x						x	x															x	x	x		x			
	Prognostic	x				x	x																	x								x								x		x			
	Research: skills			x											x																														x
Patient presentation	Incongruent/discordant									x																	x	x		x			x					x							
Personal/ communication	Dr-patient						x									x		x	x	x		x		x							x	x	x	x		x				x	x	x			
	Inter-professional					x					x					x								x									x	x						x			x		
	Recognising cues/feelings					x																		x		x		x	x							x									
Conceptual	Theory->practice					x	x									x		x	x															x		x				x					
	Knowledge boundaries																																		x		x						x		
Practical/ System	Time			x						x									x	x						x														x					
	Setting					x					x															x														x					
	Litigation										x	x																												x					
	Resources									x																x														x	x				
	educational																																			x	x								
Ethical/ Moral						x						x							x								x			x						x					x		x		

Supplemental material table 4: Matrix showing types/sub-types of uncertainty and the studies that describe them
