# Supplementary material for: What aspects of clinical uncertainty are addressed by published competencies in evidence-based medicine? A scoping review
Source: BMJ Open. 2026 Jul 31;16(7):e109013. doi: 10.1136/bmjopen-2025-109013 (PMC13435916; doi:10.1136/bmjopen-2025-109013)
Supplement: Supplementary file 6 [file bmjopen-16-7-s006.pdf]

Table. Taxonomy of Aspects of Uncertainty that Relate to EBM— Types, Properties, Modifiers and Responses

Type	Definition / Description	Sub-type	Definition / Description
**Types of Uncertainty**			
**Scientific / Technical**	**Scientific:** Uncertainty is disease-centred. Encompasses uncertainties about diagnosis, prognosis, causal explanations, and treatment recommendations.<br><br>**Technical:** Lack of theoretical information resulting in ignorance for guiding actions.	**Treatment: Evidence**	Uncertainty based on the likelihood of a treatment's outcome, or where conflicting/unclear evidence exists.
		**Treatment: Procedural**	Uncertainty in skills or judgement regarding how or when to perform a specific procedure.
		**Diagnostic**	Subjective perception of an inability to provide an accurate explanation of the patient's health problem.
		**Prognostic**	Not being certain what morbidities the patient would have or how long they would live.
		**Research: Skills**	Relating to the practice of conducting literature searches and evaluating studies.
**Patient Presentations**	Patients presenting symptoms, signs or risk factors that don't fit with the doctor's previous experience, illness scripts, or that trigger concern directly, e.g. a previous 'bad case' or risk factor.	**Incongruent**	Patterns of symptoms that didn't fit an 'illness script' or fitted with a previous case with poor outcomes.
		**Discordant**	Recognising specific features or risk factors of concern.
		**Recognising Cues / Feelings**	The recognition of the perceptual, emotional, and situational stimuli that doctors relate to uncertainty.
**Communication / Personal**	**Communication:** Inability of the physician to communicate effectively and reach a joint decision with the patient.<br><br>**Personal:** Patient-centred. Psychosocial and existential issues (relationships, impact on life goals).	**Dr–Patient**	Uncertainty arising from the communication or relationship between doctor and patient.
		**Inter-professional**	Uncertainty arising from the communication or relationship between doctor and other doctors or healthcare professionals.
**Conceptual**	Inability to apply abstract knowledge in concrete situations.	**Theory → Practice**	See definition; particularly relevant when applying evidence-based guidelines to individual patients.
		**Knowledge Boundaries**	Uncertainty about which learning approaches to use and how to manage content breadth.
**Practical / Systemic**	**Practical:** System-centred. Applies to the structures and processes of delivering care (competence, quality, responsibilities).<br><br>**Systemic:** Inability to act appropriately due to the lack of technological, technical, material and human resources, or acting outside the standards/rules of the health system.	**Time**	Instances where the acuity of a situation adds to or causes uncertainty.
		**Setting**	Uncertainty caused by a particular setting that may itself contribute to experiencing uncertainty, e.g. night shifts.
		**Resources**	Uncertainty specifically due to lack of technological, technical, material or human resources.
		**Educational**	Uncertainty arising due to specific educational settings and situations, e.g. transitioning to clinical studies.
**Ethical / Moral**	Inability to act when the person displays behaviours, attitudes and emotions inconsistent with the values/codes of society, institution, or person.	**Litigation**	Arising due to anticipation of patient complaints or being involved in legal proceedings.
**Properties of Uncertainty**			
**Probability**	Generated from the indeterminacy of a phenomenon's future outcome, such as the probability of benefit or harm of a test or treatment.		
**Ambiguity**	The lack of reliability, credibility or adequacy of information about a phenomenon of interest; includes imprecision, conflicting opinion or lack of information.		
**Complexity**	Arising from aspects of a phenomenon itself which is difficult to comprehend, e.g. numerous potential outcomes from a test/treatment or varied risk factors/signs/symptoms of a given disease.		
**Modifiers**			
**Demographic**	Factors affecting doctors' experience of uncertainty based on demographics, e.g. age, gender, or duration of experience.		
**System**	Factors affecting doctors' in-the-moment experience of uncertainty related to their working environment, including physical, situational and human factors.		
**Personal Characteristics**	Factors affecting doctors' experience of uncertainty; may include their affect or world view.		
**Response: Dimensions**			
**Cognitive**	How doctors think in response to uncertainty.		

Type	Definition / Description	Sub-type	Definition / Description
**Behavioural**	How doctors act in response to uncertainty.		
**Emotions**	How doctors feel in response to uncertainty.		
**Response: Actions**			
**Managing Available Information**	Physicians enact behaviours to gather, analyse or explain information regarding the test, treatment or proposed management plan for their patient.		
**Clinical Decision Making Strategies (CDM)**	Physicians' reasoning strategies with the aim to reduce or manage their experienced uncertainty.		
**Communicating Uncertainty**	Physicians' explanations to patients or colleagues that relate to the uncertainty, its nature, extent or strategies for dealing with it.		
